# Risk Factors of Bone Mass Loss at the Lumbar Spine: A Longitudinal Study in Healthy Korean Pre- and Perimenopausal Women Older than 40 Years

**DOI:** 10.1371/journal.pone.0136283

**Published:** 2015-08-28

**Authors:** Sungsu Kim, Jaehoon Jung, Jung Hwa Jung, Soo Kyoung Kim, Rock-Bum Kim, Jong Ryeal Hahm

**Affiliations:** 1 Division of Endocrinology, Department of Internal Medicine, Gyeongsang National University School of Medicine and Gyeonsang National University Hospital, Jinju, Republic of Korea; 2 Institute of Health Sciences, Gyeongsang National University School of Medicine, Jinju, Republic of Korea; 3 Environmental Health Center, Dong-A University, Busan, Korea; Garvan Institute of Medical Research, AUSTRALIA

## Abstract

Longitudinal studies on bone mass decline for healthy women are sparse. We performed a retrospective longitudinal study to evaluate the factor associated with bone mass changes at the lumbar spine in healthy Korean pre- and perimenopausal women over the age of 40. We examined the relation of blood tests including thyroid function tests at baseline and follow-up to the annual percentage changes in average BMD of L2-L4 (A%ΔLSBMD). Four hundred and forty-three subjects without diseases or medications pertaining to bone metabolism were analyzed. The mean A%ΔLSBMD in these subjects was -0.45%/year. Though a significant correlation was observed between the A%ΔLSBMD and age, serum thyroid-stimulating hormone (TSH) level, total cholesterol (TC) level, low-density lipoprotein cholesterol (LDL-C) level, and estimated glomerular filtration rate (eGFR) at baseline and follow-up, there was a weak correlation between A%ΔLSBMD and these variables. From multiple linear regression analyses, the percent body fat, age, serum TSH level, serum uric acid level, and the menopause at follow-up were showed to have a significant association with the A%ΔLSBMD. Unlike age, percent body fat, and menopause at follow-up, which had a negative association with the A%ΔLSBMD, serum TSH level and serum uric acid level, had a positive association with the A%ΔLSBMD. The results from our study showed that the notable risk factors of BMD loss at the lumbar spine in population of our study were advancing age, menopause, higher percent body fat, lower normal TSH, and lower serum uric acid levels.

## Introduction

Osteoporosis is a condition of decreased bone mass and quality in which the risk of fracture is increased. There is no non-invasive method of determining the bone strength and actual structure of bones. Instead, the diagnosis of osteoporosis is based on special x-ray methods called densitometry. The bone mineral density (BMD) values measured by dual-energy X-ray absorptiometry (DXA) yield a relatively accurate prediction for fracture risk [1].

In women, BMD is significantly reduced during the menopausal periods as the serum estrogen levels dramatically decrease within a short time [2]. In fact, the prevalence of primary osteoporosis has its peak among postmenopausal women. From numerous study results that investigate factors affecting bone mass in postmenopausal women, a positive association has been reported between the BMD and weight, as well as body mass index (BMI) [3–5]. After the adjustment of several factors, fat mass was recently reported to be negatively related to bone mineral content in Korean men and women [6]. In addition, numerous clinical and epidemiological studies have shown that thyroid stimulating hormone (TSH), uric acid, and lipid profile are also related to the BMD in postmenopausal women [7–13].

There is a growing body of evidence suggesting that TSH might have a beneficial effect on bone metabolism. Abe et al. revealed that bone cell precursors express TSH receptors and that TSH has defining effects on skeletal remodeling in in TSH receptor knockout mice [14]. Furthermore, in clinical observational studies low normal TSH levels were reported to be related to the low BMD, osteopenia, and osteoporosis in postmenopausal women [7,8].

Recently, observational studies reported that oxidative stress reduces BMD [15], and that BMD in postmenopausal subjects is associated with serum antioxidant carotenoids [16]. Uric acid, solely known as a byproduct of purine catabolism, once accumulated, can cause gouty arthritis and kidney stones. However, condensed water-soluble urate has been proven to have antioxidant effects [17]. In fact, a longitudinal study by Markover et al. showed that peri- and postmenopausal women with elevated uric acid level had significantly higher absolute BMD measurements at lumbar spine, and the annual rates of change in BMD at the lumbar spine being positively associated with serum uric acid levels [18].

Some large observational studies showed that elderly women who were taking statins had high BMD and low risk of fracture [19,20], and many studies have since reported that serum triglyceride levels were positively associated with BMD [10–12]. Yamaquchi et al. reported a negative correlation between plasma low-density lipoprotein cholesterol (LDL-C) level and BMD in postmenopausal women [13]. In contrast, other studies reported that there was no significant relationship between the plasma LDL-C level and the BMD in pre- and postmenopausal women [21], and that there was a positive relationship in postmenopausal women [11]. Therefore, the existence of correlation between the plasma LDL-C levels and the BMD exists is still controversial.

There were many cross-sectional studies about an association between BMD and its related factors. However, as a limitation of cross-sectional study design, it is very difficult to acknowledge the causality between them and to progressively determine their effects on the change in BMD. Since women form peak bone mass in their late 30s, bone mass gradually decreases and then shows a dramatic decline during the menopausal period [22]. However, longitudinal studies of related factors (fat percent, TSH, uric acid, and lipids) on the change in BMD in middle aged women are insufficient. Therefore, this study aimed to identify the factors that affect the changes in the BMD at the lumbar spine among healthy Korean pre- and perimenopausal women older than 40 years of age at baseline.

## Materials and Methods

### Study design and subjects

This study included 443 subjects who underwent medical checkup at the Health Promotion Center of Gyeongsang National University Hospital between 2010 and 2013. Participants who underwent medical checkup between January 2010 and December 2011 were recruited as the baseline group and were examined between January 2012 and December 2013. The participants were healthy Korean pre- and perimenopausal women older than 40 years of age at baseline, who underwent comprehensive routine health examinations with an average follow-up interval of 2 years. The reason to select women who were 40 years and older is that women experience a progressive decline in BMD, after peak bone mass is reached in their mid to late 30’s. In order to clearly observe the changes in the BMD of lumbar spine over time, subjects who had follow-up within less than 1.5 years and those with following conditions were excluded from the study: thyroid disease (thyroiditis, overt hypothyroidism and hyperthyroidism, subclinical hypothyroidism and hyperthyroidism at baseline tests, and thyroid cancer), osteoporosis, malignancy, liver disease, chronic kidney disease, diabetes, hemoglobin A1c (HbA1c) level higher than 6.5% at baseline tests, menopause at baseline, and a history of drug use that affected bone and lipid metabolisms. The study was retrospectively conducted, and the informed consent requirement for the study was exempt due to restrained database access for analysis purposes only. This study was performed with the approval of the ethical committees at Gyeongsang National University Hospital (IRB No: 2014-10-003).

### Measurements

Data were collected by reviewing self-reported questionnaires, anthropometric examination, and laboratory tests. The questionnaires included medical and fracture history, alcohol consumption, smoking, menopausal state, and age at menopause if the participant was a menopausal woman. For alcohol consumption, the participants were divided into two groups, one consisting of those who are abstinent from alcohol and the other who are not. Among subjects who drink alcohol, only few individuals (1.3%) consume more than 100 mL of alcohol a week. Of the 443 participants, 97% never smoked; therefore, smoking as a risk factor for BMD changes was excluded from the analysis.

Anthropometric data consisted of height, weight, BMI, and percent body fat. These anthropometric data, excluding height, were measured by using the impedance technique (InBody 3.0, Biospace Co., Ltd, Seoul, Korea). BMI was calculated as weight in kilograms divided by height squared in meters. Blood samples were obtained after 8 hours of overnight fasting and were used to measure fasting blood sugar (FBS), HbA1c, total cholesterol (TC), LDL-C, high-density lipoprotein cholesterol (HDL-C), triglyceride, alanine aminotransferase (ALT), uric acid, albumin, creatinine, and C-reactive protein (CRP) levels, as well as thyroid function. The TSH level was determined by using the Modular E170 (Roche, Mannheim, Germany) based on an electrochemiluminescent immunoassay. A Cobas 8000 (Roche, Mannheim, Germany) was used to measure blood uric acid, TC, triglyceride, HDL-C, and LDL-C levels by using the enzymatic colorimetric test method. The BMD measurements of the lumbar spine (L1–L4) were performed by using DXA (GE Lunar DPX-MD, Lunar Radiation Corp., Madison, WI, USA). The lumbar spine BMD represents the average BMD of L2–L4 (LSBMD). All the measurements were performed by experienced operators on the same machine at baseline and follow-up by using standardized procedures for participant positioning. The results were expressed as BMD in grams per square centimeter (g/cm^2^) by dividing the bone content by the projected area of the region scanned. The measurement precision error, expressed as coefficient of variation, was 1.4% for the lumbar spine BMD. The annual changes in the lumbar spine BMD were calculated as the difference between baseline and follow-up BMD, divided by the time intervals (year) between the two measurements. Dividing the annual changes in the lumbar spine BMD by the lumbar spine BMD at baseline and multiplying by 100 enabled us to estimate the annual percentage changes in the lumbar spine BMD (A%ΔLSBMD): the annual percentage changes in BMD (A%ΔBMD) = 100 × (follow-up BMD—baseline BMD) / baseline BMD / time intervals (year) between the two measurements.

### Statistical analyses

Continuous variables are reported in mean ± SD, and categorical variables are reported as frequencies. A paired *t* and McNemar’s test were used to analyze within-individual differences of the biological parameters and the nominal variables between the baseline and the follow-up. The Pearson correlation coefficient was used to analyze the relationship between the LSBMD and the parameters at baseline and follow-up. Moreover, the Pearson correlation coefficient was used to analyze the relationship between A%ΔLSBMD and the parameters at baseline and follow-up, mean, and differences between baseline and follow-up parameters (ΔPs). Serum ALT, triglyceride, and TSH levels were transformed common or natural logarithmically due to the distortions of their distributions that were verified by Shapiro-Wilk test. When we analyzed A%ΔLSBMD as the dependent variable, we conducted the analysis by using transformed variables as the independent variables. Multiple linear regression models are used to assess the association between the LSBMD and the parameters at baseline and follow-up, respectively. Considering multicollinearity, we excluded the variables in which the values of the variance inflation factor (VIF) was over 7. The inclusion of variables in the final model was based on the results of the stepwise regression method. In addition, multiple linear regression models are used to assess the association between the A%ΔLSBMD and the independent variables, including age, height, weight, percent body fat, alcohol, bone-related biochemical parameters at baseline, menopause at follow-up, and ΔPs that are correlated with the A%ΔLSBMD. Among the factors that showed multicollinearity (e.g., fat mass vs percent body fat, TC vs LDL-C, and FBS vs HbA1c), only the factors that had higher correlations with the A%ΔLSBMD than the other are selected. The parameters measured at baseline and follow-up, and their mean values were observed to have a similar correlation coefficient to the A%ΔLSBMD; therefore, only the values at baseline were included in the analysis. In addition, if the ΔPs had a correlation coefficient higher than 0.07 and lower than −0.07, such ΔPs were included in the analysis. To adjust the relationship between these various parameters and the A%ΔLSBMD and select the variables included in the final model, multiple linear regression was conducted as a backward method. We divided into tertiles of certain risk parameters (age, body fat percentage, TSH, and uric acid) and analyzed them in order to compare the differences in the A%ΔLSBMD between the groups by using analysis of covariance (ANCOVA). Age, percent body fat, TSH level, free thyroxine (fT4) level, uric acid level, triglyceride level, estimated glomerular filtration rate (eGFR), and differences in BMI and serum ALT levels were used as the covariance for adjustment in the ANCOVA analysis. Significant differences between the groups are based on the Bonferroni method. A p <0.05 was considered statistically significant for all the analyses. All statistical analyses were performed by using IBM SPSS version 21 statistical software for Windows (IBM Corp., Armonk, NY, USA) and R statistics version 3.2 (R Foundation for Statistical Computing, www.R-project.Org).

## Results

### General characteristics and biological parameters of the participants at baseline and follow-up

The mean age of 443 subjects was 45.0 ± 3.9 years at baseline; the follow-up duration was 2.1 years (range, 1.5–3.4 years). The number of subjects who are abstinent from alcohol was 200 (45.1%) at baseline, but decreased to 187 (42.3%) at follow-up. All the subjects were premenopausal at baseline and 76 individuals became menopausal at follow-up. In comparison to the within-individual differences between the baseline and follow-up, most parameters, including the LSBMD, weight, body fat percent, TSH level, uric acid level, HDL-C level, and LDL-C level, displayed statistically significant differences. Among these parameters, only the LSBMD, fT4 level, and eGFR showed lower mean values at follow-up than at baseline. However, the mean values for weight, percent body fat, TC, LDL-C, HDL-C, FBS, HbA1c, uric acid, and TSH levels increased with time (Table 1).

**Table 1:** General Characteristics of the Participants at Baseline and Follow-up.

Characteristics		Baseline(n = 443)	Follow-up(n = 443)	P value
**Age, year**		45.0±3.9(40–54)	47.1±3.9(42–57)	<0.001
**Menopause, n (%)**	**N**	443(100)	367(82.8)	<0.001
**Alcohol intake, n (%)**	**N**	200(45.1)	187(42.2)	0.021
**Height, cm**		159.4±5.1(141–173)	159.4±5.1(142–173)	0.528
**Weight, kg**		57.23±7.27(40.8–85.4)	57.55±7.54(43.0–86.9)	0.010
**BMI, kg/m** ^**2**^		22.53±2.68(16.8–33.8)	22.64±2.81(16.7–33.9)	0.018
**Fat mass, kg**		15.62±4.25(6.4–32.5)	16.32±4.85(6.0–36.5)	<0.001
**PBF, %**		26.94±4.65(13.9–40.3)	27.94±5.27(13.5–44.1)	<0.001
**TSH, mIU/L**		1.90±0.89(0.27–4.17)	2.04±1.11(0.05–7.94)	0.002
**Total T3, ng/dL**		105.70±15.52 (65.74–163.10)	105.49±15.18(68.33–158.40)	0.757
**Free T4, ng/dL**		1.27±0.15(0.93–1.70	1.23±0.15(0.82–1.82)	<0.001
**UA, mg/dL**		3.9±0.8(1.5–7.1)	4.1±0.9(1.8–7.9)	<0.001
**Albumin, g/dL**		4.5±0.2(3.8–5.3)	4.5±0.2(3.6–5.3)	<0.001
**FBS, mg/dL**		83.8±8.9(62–148)	85.1±11.3(62–228)	0.002
**HbA1c, %**		5.4±0.3(4.5–6.5)	5.5±0.4(4.1–7.8)	<0.001
**ALT, U/L**		16.1±7.3(6–62)	15.7±8.5(5–79)	0.378
**TC, mg/dL**		187.3±30.8(107–298)	193.0±31.1(97–312)	<0.001
**TG, mg/dL**		88.0±42.7(33–284)	90.0±49.1(29–433)	0.339
**HDL-C, mg/dL**		59.1±13.5(28–104)	61.9±14.0(22–106)	<0.001
**LDL-C, mg/dL**		115.2±28.0(30–213)	119.4±28.2(40–226)	<0.001
**Cr, mg/dL**		0.6±0.1(0.36–0.95)	0.6±0.1(0.42–0.94)	0.504
**eGFR, ml/min/1.73m** ^**2**^		101.1±20.0(61.7–208.7)	99.7±19.3(59.9–185.9)	0.036
**CRP, mg/L**		0.53±0.98(0.00–12.90)	0.69±1.23(0.10–13.00)	0.021
**Average BMD of L2~L4, g/cm** ^**2**^		1.225±0.132(0.888–1.669)	1.213±0.137(0.800–1.608)	<0.001

Values are expressed as mean±standard deviation (minium-maxium) or number (%). N, no; BMI, body mass index; PBF, percent body fat; TSH, thyroid stimulating hormone; T3, triiodothyronine; T4, thyroxine; UA, uric acid; FBS, fasting blood sugar; HbA1c, hemoglobin A1c; ALT, alanine aminotransferase; TC, total cholesterol; TG, triglyceride; HDL-C, high density lipoprotein cholesterol; LDL-C, low density lipoprotein cholesterol; Cr, creatinine; eGFR, estimated glomerular filtration rate by Cockcroft-Gault calculator; CRP, C-reactive protein; L1 BMD, lumbar1 bone mineral density. P values by paired T-test.

### Correlations between the average BMD of L2–L4 and the parameters at baseline and follow-up

**Fig 1:**
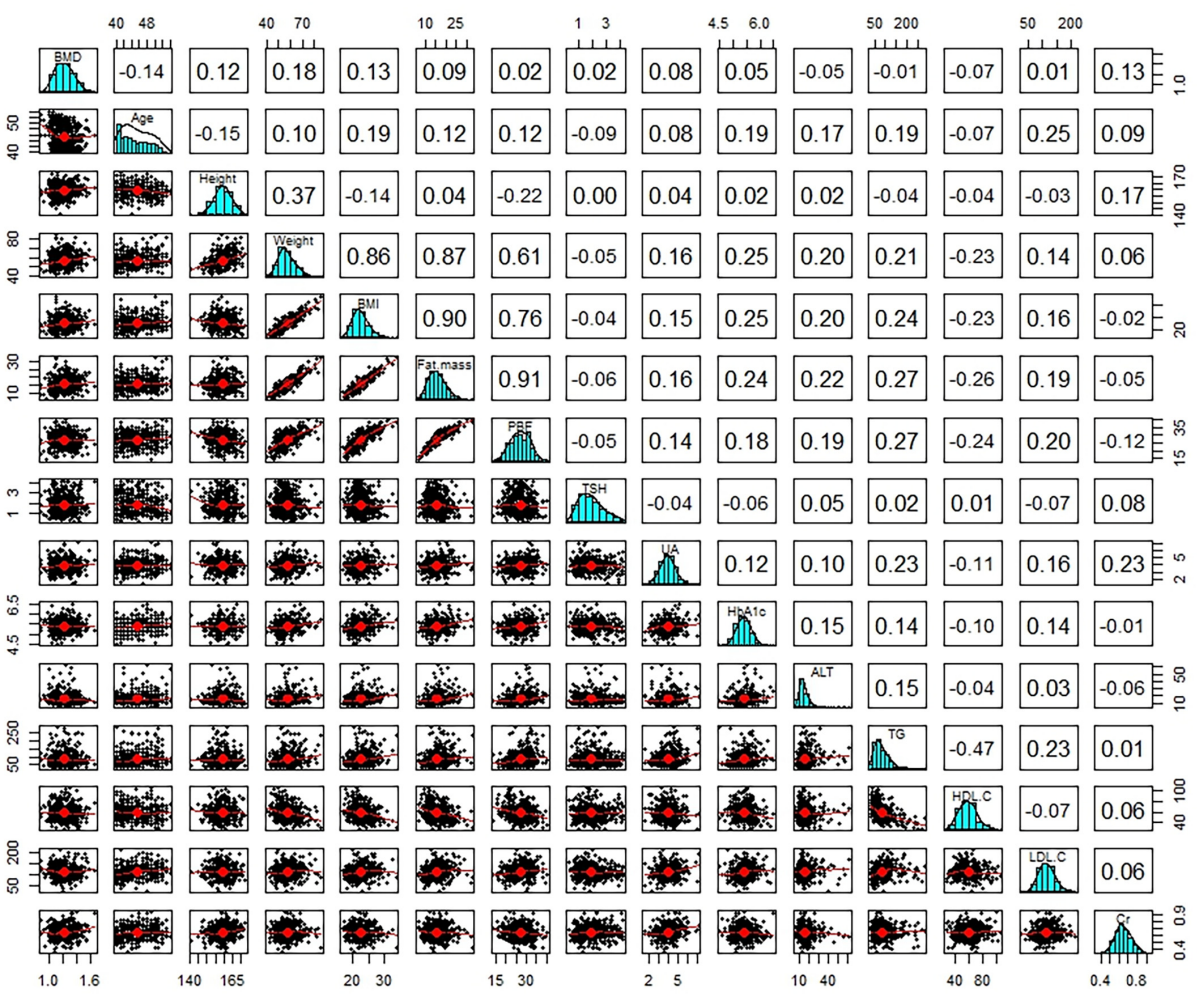
Bivariate Correlation Matrix of the Average BMD of L2-L4 with Various Parameters at Baseline. A values in square means a correlation coefficient between various parameters. A red line in scatterplot is linear regression line in two parameters. BMI, body mass index; PBF, percent body fat; TSH, thyroid stimulating hormone; T3, triiodothyronine; T4, thyroxine; UA, uric acid; FBS, fasting blood sugar; HbA1c, hemoglobin A1c; ALT, alanine aminotransferase; TC, total cholesterol; TG, triglyceride; HDL-C, high density lipoprotein cholesterol; LDL-C, low density lipoprotein cholesterol; Cr, creatinine; eGFR, estimated glomerular filtration rate by Cockcroft-Gault calculator; CRP, C-reactive protein; L1 BMD, lumbar1 bone mineral density; n, number.

**Fig 2:**
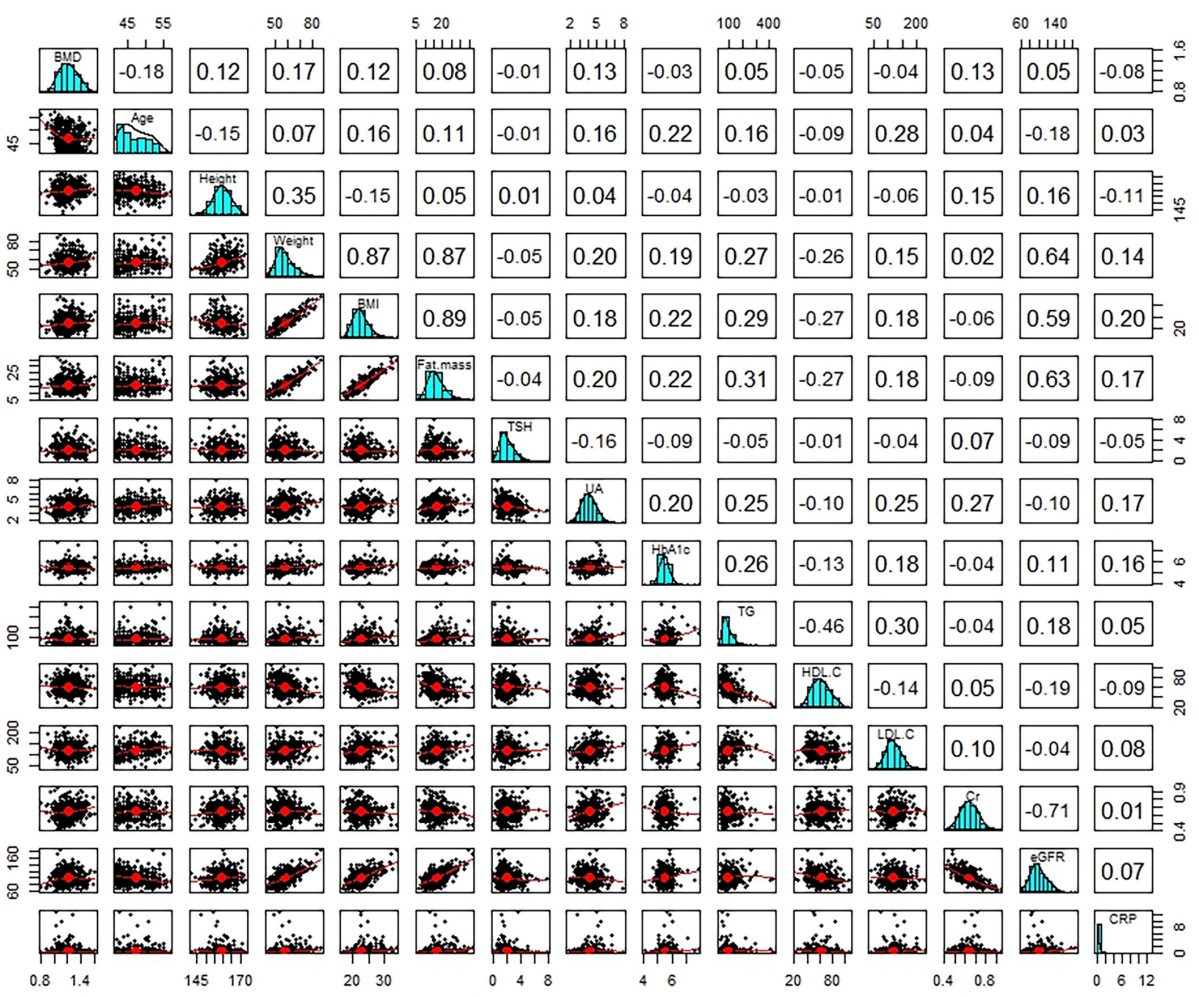
Bivariate Correlation Matrix of the Average BMD of L2-L4 with Various Parameters at Follow-up. A values in square means a correlation coefficient between various parameters. A red line in scatterplot is linear regression line in two parameters. BMI, body mass index; PBF, percent body fat; TSH, thyroid stimulating hormone; T3, triiodothyronine; T4, thyroxine; UA, uric acid; FBS, fasting blood sugar; HbA1c, hemoglobin A1c; ALT, alanine aminotransferase; TC, total cholesterol; TG, triglyceride; HDL-C, high density lipoprotein cholesterol; LDL-C, low density lipoprotein cholesterol; Cr, creatinine; eGFR, estimated glomerular filtration rate by Cockcroft-Gault calculator; CRP, C-reactive protein; L1 BMD, lumbar1 bone mineral density; n, number.

In order to determine the existence of correlation between the LSBMD and the anthropometric and biochemical parameters from baseline and follow-up, the Pearson correlation analysis was conducted. At baseline check-up, the results of the Pearson correlation analyses showed a statistically significant correlation between the LSBMD and age, height, weight, BMI, and serum creatinine level. Furthermore, the parameters that had a statistically significant correlation with the LSBMD at baseline still displayed a statistical significance at follow-up. In regards to age, as anticipated, it showed a negative correlation with the LSBMD at baseline and follow-up, with the negative correlation being strong at follow-up. As for weight, BMI, and serum creatinine level, positive correlations were observed with the LSBMD at follow-up. While the correlations being statistically significant, they were weakened when compared to those at baseline. Serum uric acid had a significantly positive correlation with the LSBMD only at follow-up. The parameters that were significantly correlated with the LSBMD were similar at baseline and follow-up, but the intensity of their correlations at follow-up showed various aspects according to each variable (Figs 1 and 2). Though several parameters were statistically significant associated with the LSBMD, these parameters had a weak correlation with LSBMD in which their correlation coefficients are between -0.35 and 0.35.

### Multiple linear regression analyses of the average BMD of L2–L4 with parameters at baseline and follow-up

. At baseline, a significant association was found between the LSBMD and weight, fat mass, age, and serum creatinine level. Weight and serum creatinine levels showed a positive association with the LSBMD, whereas age and fat mass had a negative association. At follow-up, the LSBMD had a significant association with menopause, weight, serum CRP level, serum uric acid level, fat mass, and age. Menopause, serum CRP level, fat mass, and age were negatively associated with LSBMD but were positively associated with weight and serum uric acid level. Each parameter that was related to the LSBMD was changed over about 2 years. Estimates of the fully adjusted regression models are presented in Table 2.

**Table 2:** Associations of the Average BMD of L2-L4 with Various Parameters with Parameters at Baseline and Follow-up by Multiple Linear Regression Analyses.

Parameters	UC		SC	t	Sig.	95% CI for B	Adjusted R^2^
	B	Std. Error	B				
**baseline**							0.066
** Weight, kg**	0.007	0.002	0.380	3.997	0.000	0.002, 0.005	
** Fat mass, kg**	-0.007	0.003	-0.218	-2.285	0.023	-0.012, -0.001	
** Age, year**	-0.004	0.002	-0.112	-2.403	0.017	-0.009, -0.003	
** Cr, mg/dL**	0.157	0.068	0.110	2.316	0.021	0.030, 0.294	
**Follow-up**							0.115
** Menopause at follow-up**	-0.055	0.020	-0.153	-2.789	0.006	-0.094, -0.016	
** Weight, kg**	0.007	0.002	0.366	4.049	0.000	0.003, 0.010	
** CRP, mg/L**	-0.013	0.005	-0.115	-2.498	0.013	-0.023, -0.003	
** UA, mg/dL**	0.024	0.007	0.154	3.295	0.001	0.010, 0.038	
** Fat mass, kg**	-0.006	0.003	-0.227	-2.491	0.013	-0.011, -0.001	
** Age, year**	-0.004	0.002	-0.112	-2.040	0.042	-0.008, -0.001	

SC, standardized coefficients; UC, unstandardized coefficients; B, beta; Std, standard; Sig., significance; CI, confidence interval; UA, uric acid; CRP, C-reactive protein; Cr, creatinine. Baseline predictors: weight, fat mass, age, and creatinine. Follow-up predictors: menopause at follow-up, weight, CRP, uric acid, fat mass, and age. Dependent variable: average BMD of L2-L4. P value analyzed by multiple linear regressions.

### Correlations between annual percentage changes of the averageL2–L4 BMD and the parameters

**Fig 3:**
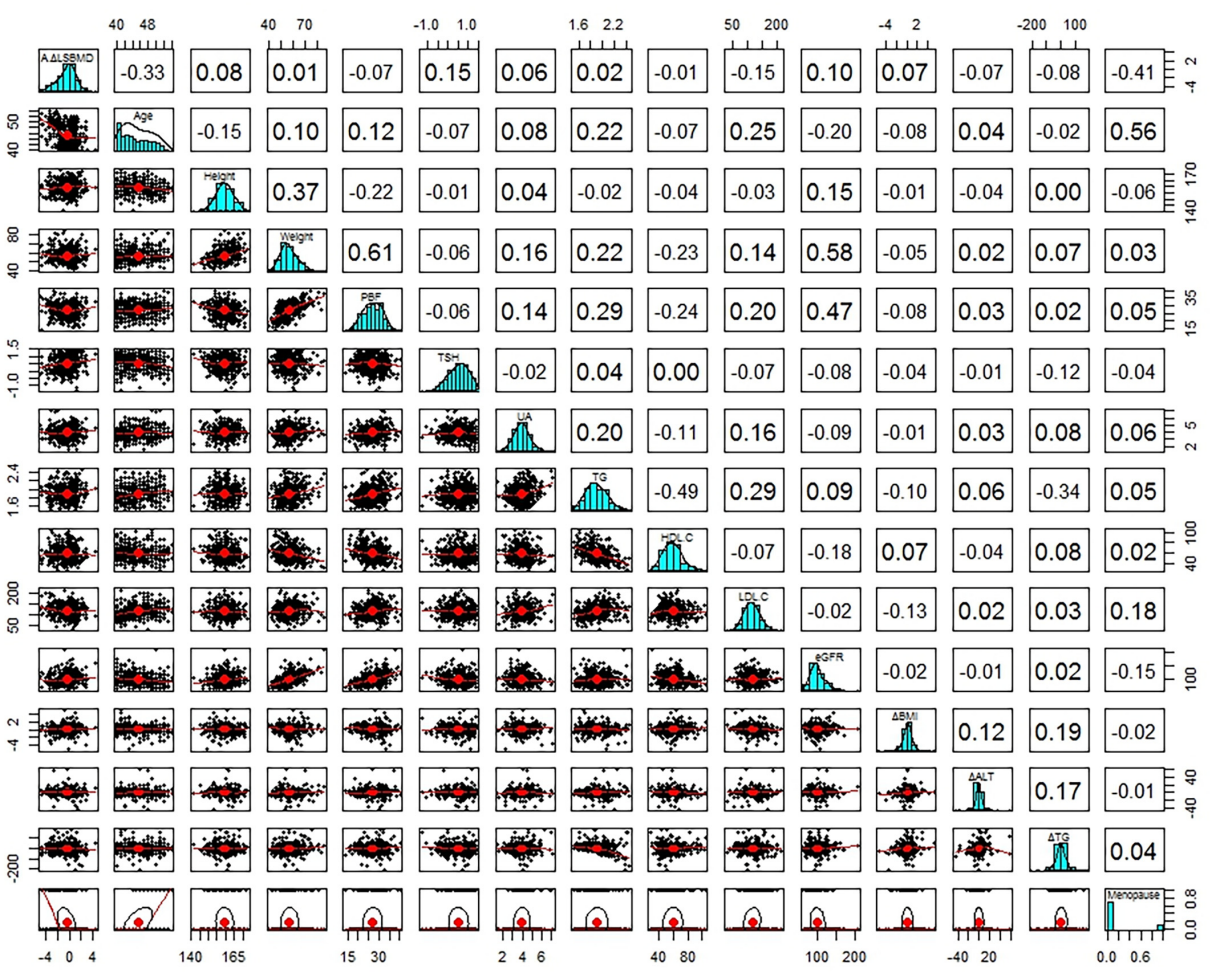
Bivariate Correlation Matrix of Annual Percentage Changes in Average BMD of L2-L4 with Various Parameters. A values in square means a correlation coefficient between various parameters. A red line in scatterplot is linear regression line in two parameters. Mean, average of values at baseline and follow-up; ΔPs, differences between baseline and follow-up parameters; BMI, body mass index; PBF, percent body fat; TSH, thyroid stimulating hormone; T3, triiodothyronine; T4, thyroxine; UA, uric acid; FBS, fasting blood sugar; HbA1c, hemoglobin A1c; ALT, alanine aminotransferase; TC, total cholesterol; TG, triglyceride; HDL-C, high density lipoprotein cholesterol; LDL-C, low density lipoprotein cholesterol; Cr, creatinine; eGFR, estimated glomerular filtration rate by Cockcroft-Gault calculator; CRP, C-reactive protein; L1 BMD, lumbar1 bone mineral density; n, number.

The mean A%ΔLSBMD in 443 subjects was −0.45%/year ± 1.15 (SD). Pearson correlation analysis was conducted to analyze a correlation between A%ΔLSBMD and parameters at baseline and follow-up, as well as the mean and differences between baseline and follow-up parameters. A significant correlations were observed between the A%ΔLSBMD and age, serum TSH level, TC level, LDL-C level, and eGFR at baseline and at follow-up. Age, TC level, and LDL-C level showed a negative correlation with the A%ΔLSBMD, whereas serum TSH level and eGFR had a positive correlation. As for HbA1c level, a positive significant correlation was observed with the A%ΔLSBMD only at follow-up. In addition, there was no significant correlation with ΔPs (Table 3 and Fig 3).

**Table 3:** Bivariate Correlation of Annual Percentage Changes in Average BMD of L2-L4 with Various Parameters.

Parameters	Correlation coefficients			
	Baseline(n = 443)	Follow-up(n = 443)	Mean(n = 443)	ΔPs(n = 433)
**Age, year**	-0.329**	-0.328**	-0.328**	-0.022
**Height, cm**	0.084	0.08	0.082	-0.032
**Weight, kg**	0.008	0.027	0.018	0.059
**BMI, kg/m** ^**2**^	-0.037	-0.01	-0.024	0.070
**Fat mass, kg**	-0.036	-0.012	-0.024	0.041
**PBF, %**	-0.067	-0.04	-0.055	0.033
**TSH** ^**†**^ **, mIU/L**	0.151**	0.137**	0.162**	0.009
**Total T3, ng/dL**	-0.028	-0.078	-0.06	-0.052
**Free T4, ng/dL**	0.035	0.059	0.055	0.025
**UA, mg/dL**	0.052	0.007	0.032	-0.058
**Albumin, g/dL**	-0.018	-0.042	-0.036	-0.021
**FBS, mg/dL**	-0.001	0.008	0.004	0.011
**HbA1c, %**	-0.069	-0.106*	-0.096*	-0.081
**ALT** ^**†**^ **, U/L**	0.009	-0.066	-0.031	-0.071
**TC, mg/dL**	-0.132**	-0.159**	-0.159**	-0.035
**TG** ^**†**^ **, mg/dL**	0.020	-0.028	-0.003	-0.084
**HDL-C, mg/dL**	-0.013	-0.01	-0.012	0.004
**LDL-C, mg/dL**	-0.152**	-0.180**	-0.179**	-0.039
**Cr, mg/dL**	-0.032	-0.02	-0.028	0.015
**eGFR, ml/min/1.73m** ^**2**^	0.098**	0.105*	0.109*	0.005
**CRP, mg/L**	-0.076	-0.059	-0.089	0.001

Mean, average of values at baseline and follow-up; ΔPs, differences between baseline and follow-up parameters; BMI, body mass index; PBF, percent body fat; TSH, thyroid stimulating hormone; T3, triiodothyronine; T4, thyroxine; UA, uric acid; FBS, fasting blood sugar; HbA1c, hemoglobin A1c; ALT, alanine aminotransferase; TC, total cholesterol; TG, triglyceride; HDL-C, high density lipoprotein cholesterol; LDL-C, low density lipoprotein cholesterol; Cr, creatinine; eGFR, estimated glomerular filtration rate by Cockcroft-Gault calculator; CRP, C-reactive protein; L1 BMD, lumbar1 bone mineral density; n, number. Parameters**†** were transformed common or natural logarithmically.

*P<0.05

**P<0.01 in bivariate correlation analyses with Pearson correlation analyses.

### Multiple linear regression analyses of annual percentage changes in the average L2–L4 BMD with parameters

Given the correlation between the A%ΔLSBMD and the various parameters, multiple linear regression analyses were conducted. In model 1 including all factors related with the A%ΔLSBMD, age, serum TSH level, serum uric acid level, and menopause at follow-up were significantly associated with A%ΔLSBMD. Unlike age and menopause at follow-up, which had a negative association with the A%ΔLSBMD, serum TSH level and serum uric acid level had a positive association. In model 2, the percent body fat, age, serum TSH level, serum uric acid level, and the menopause at follow-up was observed to have a significant association with the A%ΔLSBMD. The parameters that had a significant association with the A%ΔLSBMD in model 1 still showed a statistical significance in model 2. Furthermore, there was a significant negative association between the percent body fat level and A%ΔLSBMD in model 2. The adjusted *R* ^2^ value was 0.206 in model 1 and was 0.214 in model 2, indicating only a slight difference between the models (Table 4).

**Table 4:** Associations of the Annual Percentage Changes in Average BMD of L2-L4 with Parameters by Multiple Linear Regression Analyses.

Parameters	UC		SC	t	Sig.	95% CI for B	Adjusted R^2^
	B	Std. Error	B				
**Model 1**							0.206
**Age, year**	-0.053	0.024	-0.128	-2.200	0.028	-0.099, -0.006	
**Height, cm**	-0.006	0.018	-0.021	-0.348	0.728	-0.041, 0.028	
**Weight, kg**	0.013	0.017	0.064	0.811	0.418	-0.019, 0.046	
**PBF, %**	-0.037	0.023	-0.112	-1.588	0.113	-0.082, 0.009	
**TSH** ^**†**^ **, mIU/L**	0.407	0.133	0.135	3.074	0.002	0.147, 0.668	
**Total T3, ng/dL**	-0.005	0.004	-0.050	-1.103	0.271	-0.013, 0.004	
**Free T4, ng/dL**	0.844	0.478	0.081	1.766	0.078	-0.096, 1.785	
**UA, mg/dL**	0.193	0.084	0.106	2.304	0.022	0.028, 0.358	
**Albumin, g/dL**	-0.358	0.297	-0.054	-1.204	0.229	-0.943, 0.226	
**HbA1c, %**	-0.081	0.243	-0.015	-0.334	0.738	-0.559, 0.396	
**ALT** ^**†**^ **, U/L**	0.358	0.490	0.037	0.731	0.465	-0.606, 1.322	
**TG** ^**†**^ **, mg/dL**	0.736	0.479	0.090	1.535	0.126	-0.206, 1.677	
**HDL-C, mg/dL**	0.003	0.006	0.025	0.498	0.618	-0.008, 0.014	
**LDL-C, mg/dL**	-0.004	0.003	-0.067	-1.389	0.166	-0.009, 0.002	
**eGFR, ml/min/1.73m** ^**2**^	0.005	0.005	0.068	1.122	0.263	-0.004, 0.014	
**Alcohol intake**	-0.025	0.136	-0.008	-0.188	0.851	-0.292, 0.241	
**ΔHbA1c, %**	-0.257	0.280	-0.040	-0.919	0.359	-0.807, 0.293	
**ΔBMI, kg/m** ^**2**^	0.096	0.066	0.064	1.451	0.147	-0.034, 0.226	
**ΔALT, U/L**	-0.008	0.008	-0.049	-0.999	0.318	-0.025, 0.008	
**ΔTG, mg/dL**	-0.001	0.002	-0.041	-0.828	0.408	-0.005, 0.002	
**Menopause at follow-up**	-1.281	0.210	-0.318	-6.092	<0.001	-1.695, -0.868	
**Model 2**							0.214
**Age, year**	-0.043	0.023	-0.105	-1.910	0.047	-0.087, -0.001	
**PBF, %**	-0.031	0.017	-0.094	-1.794	0.042	-0.064, -0.002	
**TSH** ^**†**^ **, mIU/L**	0.405	0.130	0.134	3.111	0.002	0.149, 0.664	
**Free T4, ng/dL**	0.754	0.462	0.072	1.632	0.103	-0.154, 1.663	
**UA, mg/dL**	0.185	0.080	0.101	2.298	0.022	0.027, 0.343	
**TG** ^**†**^ **, mg/dL**	0.746	0.385	0.091	1.936	0.053	-0.011, 1.503	
**eGFR, ml/min/1.73m** ^**2**^	0.007	0.004	0.094	1.798	0.073	-0.001, 0.015	
**ΔBMI, kg/m** ^**2**^	0.096	0.065	0.065	1.495	0.136	-0.030, 0.223	
**ΔALT, U/L**	-0.013	0.007	-0.076	-1.779	0.076	-0.028, 0.001	
**Menopause at follow-up**	-1.316	0.208	-0.327	-6.336	<0.001	-1.725, -0.908	

SC, standardized coefficients; UC, unstandardized coefficients; B, beta; Std, standard; Sig., significance; CI, confidence interval; BMI, body mass index; PBF, percent body fat; TSH, thyroid stimulating hormone; T3, triiodothyronine; T4, thyroxine; UA, uric acid; HbA1c, hemoglobin A1c; ALT, alanine aminotransferase; TG, triglyceride; HDL-C, high density lipoprotein cholesterol; LDL-C, low density lipoprotein cholesterol; eGFR, estimated glomerular filtration rate by Cockcroft-Gault calculator; ΔPs, differences between baseline and follow-up parameters. Parameters† were transformed common or natural logarithmically. Model 1 predictors: menopause at follow-up, albumin, ΔALT, TSH, HDL-C, height, ΔBMI, alcohol, uric acid, T3, ΔHbA1c, A1c, fT4, ΔTG, LDL-C, eGFR, ALT, age, PBF, TG, and weight. Model 2 predictors: menopause at follow-up, ΔALT, TSH, ΔBMI, uric acid, fT4, eGFR, age, PBF, and TG. Dependent variable: annual percentage changes in average BMD of L2-L4. P value analyzed by multiple linear regressions.

**Fig 4:**
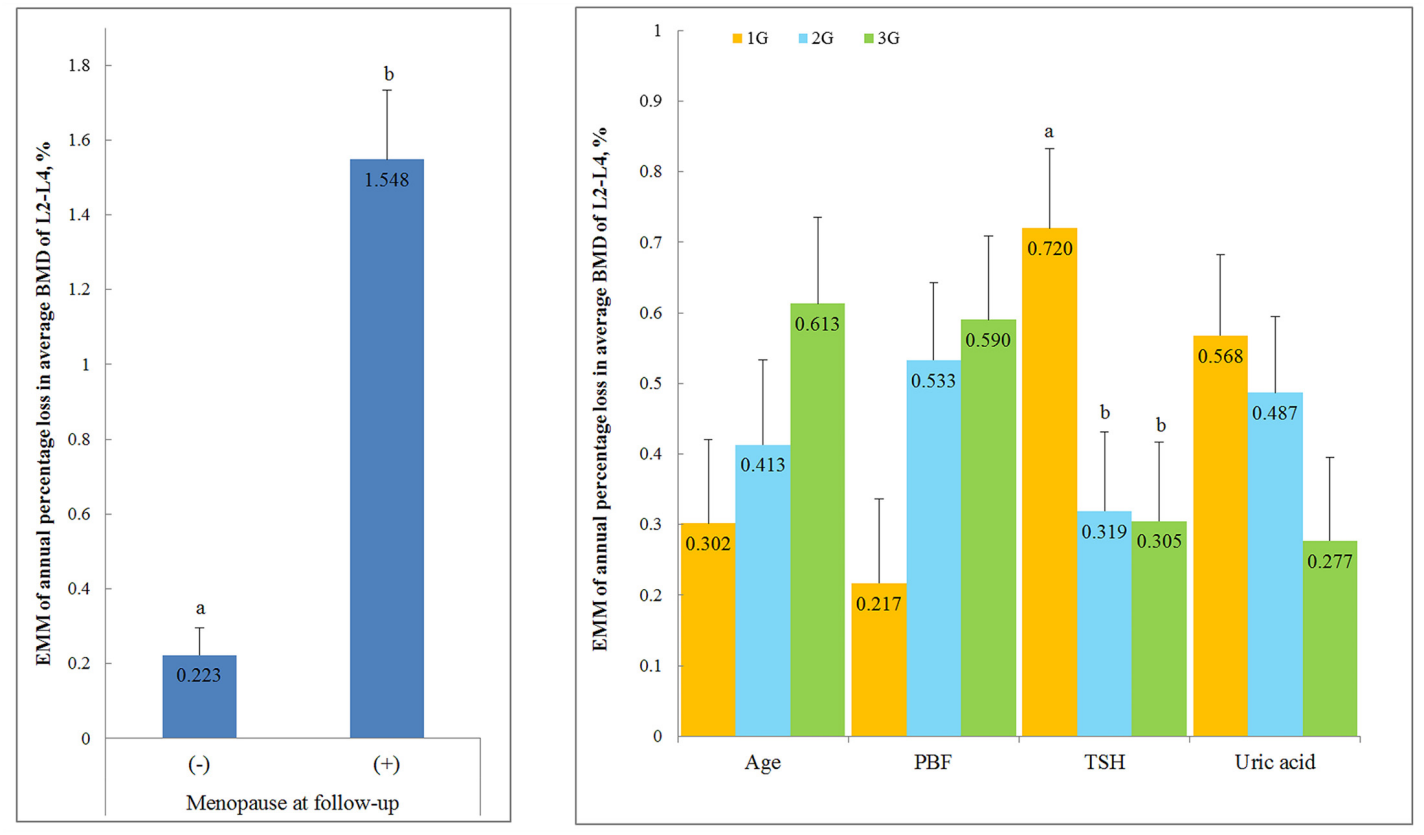
Estimated Marginal Means of Annual Percentage Loss in Average BMD of L2-L4 for Selected Risk Factors. Estimated marginal means in SPSS general linear model are adjusted for the covariates. Covariates appearing in the model are evaluated at the following values: age, percent body fat, TSH, free T4, uric acid, TG, eGFR, ΔBMI, and ΔALT. The subjects were split into three equally sized groups (tertiles) according to age (40~42, 43~46, 47~54, years), percent body fat (13.9~24.7, 24.8~29.5, 29.6~40.3, %), serum TSH levels (0.27~1.38, 1.39~2.19, 2.20~4.17, mIU/L), and uric acid (1.5~3.5, 3.6~4.2, 4.3~7.1, mg/dL) in the baseline check-up. a,b same or different letters denote no or significant differences between the groups. Significant differences between the groups are based on Bonferroni method. EMM, Estimated marginal means.

To investigate the A%ΔLSBMD affected by each parameter, the ANCOVA was used to compute the estimated marginal mean (EMM). The EMM of A%ΔLSBMD in the women who were found to be menopausal at their second visit was -1.584%, which proved that the menopausal women experienced rapid bone mass loss compared to their counter parts. The parameters such as age, percent body fat, serum TSH level, and serum uric acid level, which were significantly associated to the A%ΔLSBMD in model 2, were assigned into three groups (tertiles). The estimated marginal mean of A%ΔLSBMD among the subjects of the group with serum TSH levels 0.27–1.38 mIU/L was -0.720%/year. Results of the ANCOVA analyses showed that low normal TSH levels (0.27–1.38 mIU/L) were significantly associated with lumbar spine BMD loss compared to the subjects with high serum TSH levels (Fig 4).

## Discussion and Conclusions

We found that age, menopause, and higher percent body fat had significantly negative association with A%ΔLSBMD in Korean healthy pre-and perimenopausal women at baseline. Meanwhile, higher serum TSH and uric acid levels were associated with lesser bone mass loss at the lumbar spine.

From this study, age and menopause were negatively associated with LSBMD and A%ΔLSBMD. It is well-known that age and menopause have strong associations with BMD reduction in women and are inevitable risk factors for osteoporosis [22–27]. As the rate of BMD loss increase with advancing age in elderly women [23,24], we showed that advancing age in middle aged women is also a risk factor of decline in LSBMD and A%ΔLSBMD. However, aging in middle aged women is a weaker risk factor than in elderly women. The dramatic reduction in BMD during the menopausal period has been mainly attributed to estrogen deficiency [2,28]. The major physiological effects of estrogen on bone remodeling are to prevent the bone resorption by reducing the formation and activation of osteoclasts [29]. Pouilles et al. grouped women aged 45 to 66 years into pre-, peri-, and postmenopausal groups according to their menopausal status [26]. Their study showed that the loss of lumbar BMD mostly occurred at perimenopausal period and within 3 years after menopause [26].

In the cross-sectional analyses of LSBMD and various parameters measured at baseline and follow-up, the lower LSBMD was observed as fat mass increased. Furthermore, a negative association was observed between percent body fat and the A%ΔLSBMD. Although no clinical study targeting women older than 40 years has yet examined the relationship between the A%ΔLSBMD and percent body fat, numerous cross-sectional studies demonstrated the association between BMD and percent body fat [6,30–32]. Kim et al. took pre- and menopausal Korean women older than 40 years and demonstrated a negative association between fat mass and bone mineral content after for age and weight adjustment in their cross-sectional study [6]. Meanwhile, the study conducted from premenopausal and postmenopausal Thai women aged 40 to 49 years reported that fat mass was rather positively associated with bone mass in the premenopausal women [30]. Such conflicting results are due to the different selections of study design, targeted sample population, sample structure, and covariates. As fat mass has a highly positive correlation with weight, it is not easy to separate a mechanical load effects and negative effects on bone metabolism caused by excessive fat tissues in a statistically distinct manner. Many clinical cross-sectional studies reported that weight is positively associated with BMD at lumbar spine in pre- and postmenopausal women [5,33]. Furthermore, Framingham osteoporosis study (FOS) demonstrated there was bone mass loss in association with being underweight and having great weight loss from elderly men and women older than 67 years [33]. In our study, weight displayed a positive association with the LSBMD at baseline and follow-up, which was statistically significant after precise adjustment. It had no actual association with the A%ΔLSBMD, however. The population of our study consisted of younger women than those of FOS, suggesting that a mechanical load in weight has more association with the peak bone mass formation in the late 30s, rather than with having protective effects on lumbar bone mass loss after the 40s.

It is well known that hyperthyroid status is associated with reduced BMD. It was reported that serum TSH have a positive association with BMD at the lumbar spine and femoral neck in healthy postmenopausal women [7,8]. However, Grimnes et al. showed that it had no association with BMD within a normal serum TSH level range [34]. In addition, Lin et al. reported not only the absence of a statistically significant correlation between BMD and serum TSH level but also a negative correlation between BMD and serum total T4 level in the postmenopausal Taiwan healthy women [35]. Moreover, according to a prospective study with menopausal women older than 65 years, lower serum TSH level had no association with lower BMD, as well as accelerated bone loss [36]. We found that TSH level was not associated with lumbar spine BMD, but it had significantly positive association with the A%ΔLSBMD. Although results are conflicting in previous studies, it is not appropriate to compare with those studies due to differences of targeted sample population, sample structure, and covariates. It is difficult to distinguish the effects of TSH and thyroid hormone from those of bone metabolism through a retrospective study, as thyroid hormone and TSH have a biologically reciprocal relationship. Though it seems premature at present to draw a strong conclusion, we showed that lower normal TSH levels were associated with rapidly lumbar BMD loss in healthy middle aged women.

In the case of uric acid level, LSBMD measurements had significantly positive association with the serum uric acid level, and high uric acid level at baseline had a protective effect on the lumbar spine BMD loss, which is consistent with the results of previous study by Makovey J. [18]. Increased serum uric acid level is often observed among individuals with metabolic syndrome; that is, higher serum uric acid level is correlated to higher fat mass, serum triglyceride, TC level, and LDL-C level and to lower HDL-C level [18,37]. In Pearson correlation analysis, TC and LDL-C level were negatively and significantly correlated with the A%ΔLSBMD and uric acid level had not significant correlation. After the adjustment, uric acid level showed positive and significant association with the A%ΔLSBMD. Despite the fact that beneficial effects of uric acid on bone mass loss have been associated with weight-bearing, antioxidant or bystander effects, we showed that the its association with bone mass loss was unequivocally evident even after adjustment of weight and biochemical parameters that other studies did not attempt to adopt.

There are a few limitations in this study. First, although we examined a lot of factors associated with LSBMD and A%ΔLSBMD, the values of adjusted R^2^ in our regression models were so low at approximately 6%~20% that the explanatory power of factors as to bone loss and BMD was low. Bone loss and BMD can be associated with other factors that were not investigated in this study. Factors such as calcium and vitamin D intake, serum 25-OH vitamin D level, and physical activity were not included in the analyses. Second, we had only two BMD measurements in a relatively short time interval. Several points of BMD measurements over time can be more appropriate in order to identify the risk factors for BMD loss. Third, included subjects were not from the general community but from the single tertiary medical hospital, and were willing to health check-ups. Finally, the measurement of lumbar spine BMD can be affected by degenerative changes which could artificially elevate the determinants of BMD. We didn’t have any tests that can verify whether the degenerative change of lumbar spine was accompanied. Thus, without considering such a bias, the results of this study cannot be generalized and applied to the population that our study presented. In spite of these limitations, we conducted investigations and analyses for various anthropometric and biochemical measurements, and their changes related to LSBMD and A%ΔLSBMD in middle aged women. To the best of our knowledge, we report for the first time that percent body fat and serum TSH level are significantly associated with the A%ΔLSBMD in healthy pre-and perimenopausal women older than 40 years at baseline.

The results of our study showed that risk factors of BMD loss at the lumbar spine in population of our study were advancing age, menopause, higher percent body fat, lower normal TSH, and lower serum uric acid levels. However, further studies are needed to determine the precise mechanism underlying the association between BMD loss and percent body fat, serum TSH, and uric acid level.
